# Systematic Review of Cholinesterase Inhibitors on Cognition and Behavioral Symptoms in Patients of Chinese Descent with Alzheimer’s Disease, Vascular Dementia, or Mixed Dementia

**DOI:** 10.3390/geriatrics2030029

**Published:** 2017-08-27

**Authors:** Ka-Chun Leung, Victor Li, Yuey-Zhun Ng, Tsz-Tai Chan, Richard S. Chang, Roger Y. Wong

**Affiliations:** 1 LKS Faculty of Medicine, University of Hong Kong, Hong Kong, China; trumanl@hku.hk (K.-C.L.); u3505468@connect.hku.hk (V.L.); u3505541@connect.hku.hk (Y.-Z.N.); tsztai@connect.hku.hk (T.-T.C.); 2 Division of Neurology, Department of Medicine, Queen Mary Hospital, Hong Kong, China; richardcsk@gmail.com; 3 Dean’s Office, Faculty of Medicine and Division of Geriatric Medicine, Department of Medicine, Faculty of Medicine, University of British Columbia, Vancouver, BC V6T 1Z4, Canada

**Keywords:** Chinese, dementia, cholinesterase inhibitors, cognition, behavior

## Abstract

Cholinesterase inhibitors (ChEIs) are the primary pharmacologic treatment for dementia. Their efficacy in patients of Chinese descent is not well described. We reviewed how ChEIs could affect cognition and behavioral and psychological symptoms (BPSD) in Chinese patients with Alzheimer’s disease (AD), vascular dementia (VaD), or mixed (AD with vascular component) dementia. MEDLINE, PsycINFO, EMBASE and CINAHL were systematically searched for controlled trials of ChEIs, including donepezil, galantamine, and rivastigmine, for Chinese patients with AD, VaD, or mixed dementia. Outcomes for cognition and BPSD were extracted for discussion. Fifty-four studies were identified. While one larger study found that dementia patients of Chinese descent treated with ChEIs had significantly higher mean Mini-Mental State Examination (MMSE) score, other studies showed no significant difference. Evidence on BPSD after use of ChEIs was also conflicting. ChEIs may be effective in improving cognition among patients of Chinese descent with dementia. Further studies are needed to examine the possible effects of ChEIs on BPSD in Chinese patients with dementia in view of the small number of studies and limitations in their methodologies.

## 1. Introduction

Dementia is a condition characterized by a progressive decline in cognitive and functional abilities. With an estimated global prevalence of 35.6 million [1], dementia is the leading cause of disability in the elderly population in many countries [2,3]. Patients with Alzheimer’s disease (AD) have reduced cerebral production of acetylcholine and impaired cortical cholinergic function. Hence, augmentation of cholinergic activity therapeutically may alleviate cognitive decline in dementia [4]. Cholinesterase inhibitors (ChEIs) including donepezil, galantamine, and rivastigmine, which raise acetylcholine levels in the brain via the inhibition of acetylcholinesterase, are considered first-line pharmacotherapy for dementia. While current evidence supports the efficacy of ChEIs in improving cognition, most of the studies were conducted in Europe and North America, with the majority of subjects being Caucasians. No systematic review has been done to specifically appraise evidence to decide whether ChEIs are efficacious in Chinese patients with dementia.

In addition to cognitive decline, dementia patients may also experience various neuropsychiatric symptoms including agitation, aberrant motor behavior, anxiety, elation, irritability, depression, apathy, disinhibition, delusions, hallucinations, and sleep or appetite changes. This heterogeneous group of clinical phenomena is collectively referred to as behavioral and psychological symptoms of dementia (BPSD) [5], and it is estimated that more than 90% of dementia patients will experience at least one episode of BPSD [6]. A cross-sectional study conducted in Shanghai also revealed that more than 50% of community-living Chinese dementia patients had at least one episode of BPSD [7]. Similar to the cholinergic deficiency hypothesis for cognitive decline, it is suggested that BPSD in dementia can be related to a decline in acetylcholine in brain regions regulating behavioral and emotional responses, such as the limbic system. ChEIs can potentially be beneficial in alleviating BPSD in patients with dementia [8]. Galantamine, in addition to its effect on cholinesterase inhibition, also enhances dopaminergic neurotransmission via the allosteric potentiation of nicotinic acetylcholine receptors [9]. It was shown in a study conducted in Thailand that galantamine improved psychotic, behavioral, and psychological symptoms in patients with AD [10]. Despite the prevalence of BPSD in both Caucasian and Chinese dementia patients, there is still a lack of consensus with regard to the efficacy of ChEIs in treating BPSD, since although a number of reviews and individual studies have demonstrated the reduction of BPSD [6,11,12], other studies have suggested otherwise [13,14,15,16].

Cognitive decline and BPSD not only constitute major sources of distress and poor quality of life to both dementia patients and their caregivers, but dementia, as a public health priority, also entails significant societal and economic cost [17]. The Chinese population, with its 8.36 million dementia patients as of 2012, faces a particularly heavy disease burden as the prevalence is expected to double in 20 years [18]. Finding an effective management strategy for dementia would therefore carry significant implications in patient management and public health policies. However, pharmacogenetic studies suggest that response to ChEIs may be different in the Chinese population. For example, CYP2D6, whose pleomorphism is partially responsible for variable clinical responses to ChEIs [19], demonstrates different allelic distributions in Chinese and Caucasian populations [20]. Whether such biological differences translate into altered treatment response in Chinese dementia patients remains unclear. By conducting this systematic review, we would like to delineate the effects of ChEIs on the cognition and BPSD of Chinese patients with dementia.

## 2. Materials and Methods

### 2.1. Literature Search

Four databases, MEDLINE, PsycINFO, EMBASE and Cumulative Index to Nursing and Allied Health Literature (CINAHL), were searched with the assistance of a medical librarian. The exact search strategies are available upon request.

### 2.2. Inclusion and Exclusion Criteria

The articles retrieved from the databases were further processed by two independent reviewers. Those that were (a) published from 1 March 2000 to 1 March 2015; (b) original studies on ChEIs with the presence of a control group, e.g., randomized controlled trials, open-label trials, and cohort studies. ChEIs refer to donepezil, galantamine, or rivastigmine; (c) sampling patients who were Chinese of Far East Origin and (d) who were diagnosed with AD, VaD, or mixed dementia were selected for our study. Head-to-head trials and studies using combination regimens were excluded. Chinese of Far East Origin, a Medical Subject Heading (MeSH), accommodated patients from the countries and regions of China, Hong Kong, Japan, Korea, Macau, Mongolia, and Taiwan. Articles written in English and Chinese were included in the review. In view of the paucity of studies, no restriction was placed on blinding or sample size.

### 2.3. Data Collection and Quality Assessment

The following information was extracted from the selected studies: the country or region in which the studies were carried out, study designs, sample sizes, numbers of center, study and follow-up durations, diagnostic criteria used for diagnosing dementia, inclusion and exclusion criteria of recruitment, age, sex, ethnicity and home country of patients, whether the patients were institutionalized, and source of funding of the studies. For quality assessment, the Cochrane Risk of Bias Tool was used [21]. The tool was developed for assessing randomized trials and included six components, namely sequence generation, concealment of treatment allocation, blinding of participants and personnel, incomplete outcome data, selective reporting, and other sources of bias. Outcomes were cognition and BPSD, assessed by Mini Mental State Examination (MMSE) and Neuropsychiatric Inventory (NPI), respectively.

### 2.4. Discrepancy Resolution

For discrepancies between any two reviewers during the process of study selection and data collection, a panel review involving all available research team members was carried out and consensus was achieved.

## 3. Results

### 3.1. Literature Search Flow

**Figure 1:**
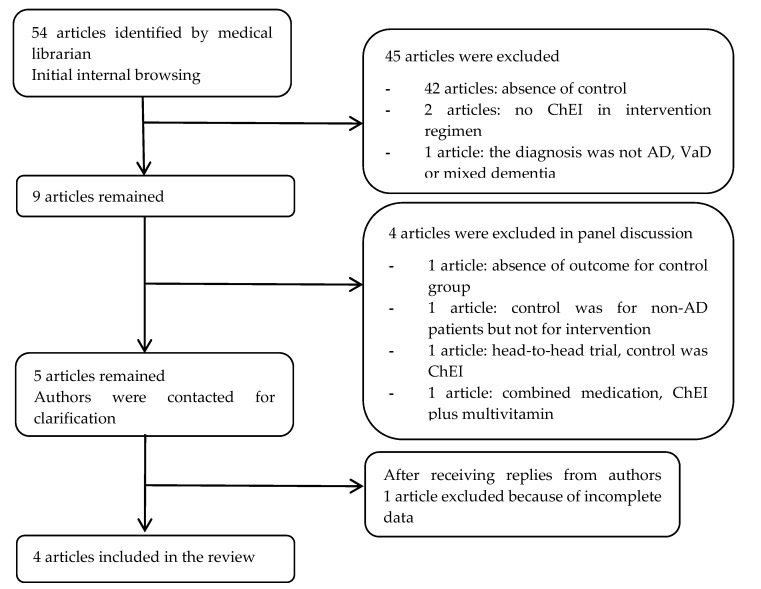
Flow diagram of article inclusion for review. ChEI, cholinesterase inhibitor; AD, Alzheimer’s disease; VaD, vascular dementia.

Fifty-four studies were initially identified and reviewed by two independent reviewers (Figure 1). Forty-two articles were excluded due to the absence of a control group. Cholinesterase inhibitor was not part of the intervention regimen in two of the remaining articles. One remaining study was excluded as only patients with mild cognitive impairment were recruited as subjects. Nine studies remained and disagreement in the inclusion of four of the studies were submitted for panel discussion. All four studies were subsequently excluded due to the absence of outcome for the control group, inappropriate intervention, or inappropriate control group. Authors of the five selected studies were contacted for clarification of data. One study was then removed due to incomplete data and no appropriate reply from the corresponding author. Four studies were included according to the search strategy in the systematic review. Two studies were randomized controlled trials (RCTs) and the other two were cohort studies.

### 3.2. Characteristics of Trials and Participants

Different cholinesterase inhibitors, namely galantamine, rivastigmine, and donepezil, were used in these studies. Baseline MMSE and NPI scores were largely similar in the studies conducted by Chu et al. and Mok et al. [13,14]. The treatment group had significantly higher MMSE, lower clinical dementia rating (CDR) stages, and less psychotic symptoms than the control group in the cohort study by Fuh et al. [22]. The mean study duration ranged from 12 weeks to 29 months. The compliance/completion rate ranged from 77.5% to 98.9% in the reported studies [13,14,23] (Table 1).

Three studies reported cognition change measured by MMSE score with heterogenous results [13,14,23]. A randomized controlled trial (RCT) involving 89 patients with mild to moderate Alzheimer’s disease demonstrated significantly higher MMSE score in the donepezil group when compared to the control after 12 weeks of treatment [23]. Another RCT conducted by Mok et al. enrolled 40 patients with subcortical vascular dementia and found no effect of galantamine treatment on cognition [13]. In a cohort study by Chu et al., 42 patients with mild to moderate Alzheimer’s disease who received galantamine treatment were compared to 19 patients in a historical cohort [14]. Two-year follow-up did not demonstrate any statistically significant change in cognition. A longitudinal observational study by Fuh et al. evaluated the effect of ChEIs on cognition by calculating the probability of transiting to higher CDR stages [22]. Use of ChEIs was associated with significantly lower chance of transiting to higher stages. Nonetheless, patients in the treatment group had a milder disease, which may have confounded the apparent finding.

Regarding BPSD, the cohort study by Chu et al. showed that there was a statistically significant increase in NPI score in the intervention group, while no significant change was seen in the control group [14]. The authors therefore concluded that galantamine may worsen BPSD. However, Mok et al. noticed a trend of improved BPSD in the galantamine group by four points of the NPI score, with improvement being most notable in irritability and aberrant motor behavior, although no statistical significance was achieved [13].

### 3.3. Assessment of Study Quality

**Figure 2:**
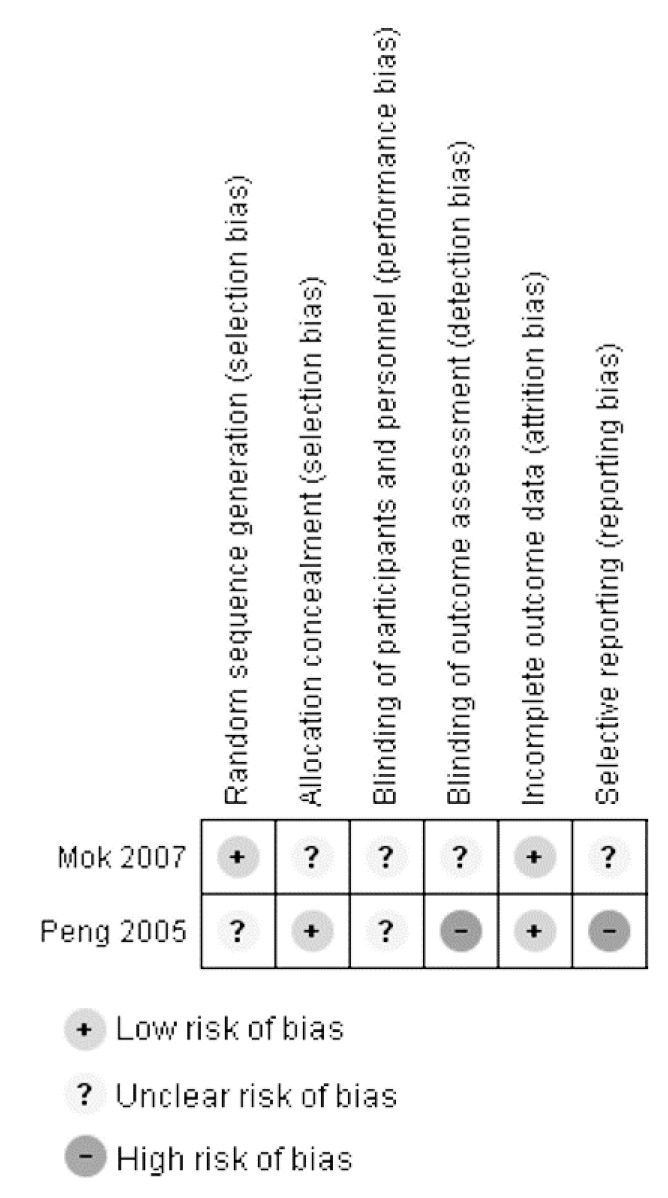
Quality assessment of included RCTs. Template based on and produced with the software RevMan version 5.3 [24].

Quality assessment was performed for the two RCTs by Mok et al. and Peng et al. [13,23] (Figure 2). With regard to random sequence generation, Mok et al. employed a computer program generated code whereas details of randomization were not stated by Peng et al. Method of allocation concealment was not mentioned in either study. Risk of selection bias cannot be ascertained. Blinding of participants, personnel, and outcome assessors was also inadequately described by Mok et al. In the study by Peng et al., while blinding of participants was performed by the provision of placebo and donepezil tablets that were identical in physical appearance, it is uncertain whether study personnel were blinded. The lack of blinding of outcome assessors in Peng et al. also renders their study at a high risk of detection bias. Both studies had near complete data, and reasons were clearly stated for missing entries. The risk of reporting bias cannot be determined in the study by Mok et al. due to the lack of available protocol, but the risk was high in Peng et al. due to the reporting of measures that were not pre-specified.

## 4. Discussion

This systematic review evaluates the effects of ChEIs on cognition and BPSD in Chinese patients with dementia. Regarding cognition, while two small studies were unable to demonstrate significant effect, one larger trial showed that donepezil was associated with an increase in MMSE score. An observational study also found a reduced chance of progression associated with ChEIs. Evidence on BPSD was conflicting. One cohort study suggested that ChEIs are linked to an increase in NPI score but another RCT suggested a trend towards improved BPSD.

The possible improvement of cognition was compatible with previous systematic reviews of RCTs performed in Caucasian populations [12,15,25,26], which favored ChEIs over placebo with mean changes in MMSE ranging from 0.74 to 1.84 points. The effect of ChEIs on BPSD is more debatable in the current literature. Some studies have suggested that ChEIs may improve BPSD, but the results should be interpreted with caution. For instance, Rodda, Morgan and Walker examined evidence on using ChEIs to treat BPSD in a systematic review. Four out of 14 trials identified demonstrated statistically significant decreases in NPI in treatment groups [27]. Donepezil was shown to decrease NPI in a meta-analysis of three trials, but one of the three studies showed a trend towards worsened BPSD [12]. Other studies could not demonstrate the effect of ChEIs on NPI score. A systematic review of rivastigmine identified three studies using NPI as the outcome and could not detect a difference between the treatment and control groups [15]. Another systematic review identified three trials reporting the effect of galantamine on NPI at six months, in which significant reduction was found in one study using 16 mg per day but not in studies using higher doses, raising doubts on the dose-response relationship [26]. Some studies even hinted at the possibility of ChEIs leading to worsened BPSD, but no unequivocal conclusion could be drawn. An RCT in 208 nursing home residents with a mean age of 86 years was conducted to examine the effect of donepezil on BPSD. It was found that donepezil was associated with a trend towards worse outcomes in 11 of the 12 behaviors assessed in the Neuropsychiatric Inventory-Nursing Home Version (NPI-NH), though the overall difference of the NPI-NH score was not statistically significant [28]. In an open-label study of 101 patients under primary care, Rockwood et al. found that around 20% of clinicians and caregivers reported worsened mood and delusion after using donepezil, although a similar proportion of subjects reported improvement [29]. The conflicting evidence in patients of Chinese descent echoes the continuing debate of ChEIs’ effect on BPSD in Caucasian patients. The conflicting evidence with regard to the effects of ChEIs on BPSD could be due to inherent biological differences between patient cohorts from different study populations, which may influence the specific subtype of BPSD and hence the response to ChEIs. One such example may be related to differences in apolipoprotein E (APOE) genotypes, which was suggested to correlate, albeit inconclusively, with different neuropsychiatric endophenotypes such as apathy and agitation [30]. It is unclear whether this difference in APOE genotypes was present across different patient cohorts in the included studies.

Currently, no pharmacological mechanism has been proposed to explain why ChEIs may worsen BPSD. With in vivo study suggesting that cholinergic deficit is positively correlated with behavioral symptoms in AD patients, the finding of worsened BPSD associated with ChEIs is surprising [31]. However, how the severity of BPSD and predominating symptoms vary along the course of the disease progression may shed light on this observation. Longitudinal series found that the NPI score gradually decreases as dementia progresses [32,33]. Multiple studies support the observation that mood and anxiety problems become more severe, whereas apathy increases [34]. Prominent depressive symptoms in the early stage of dementia were attributed to preserved insight into cognitive deficit and loss of independence [35]. It is therefore possible that apparent worsening of BPSD may be secondary to improved cognition brought by ChEI treatment. Scales delineating different subsets of BPSD of different suspected origins, such as those caused by partially preserved or improved cognition and those caused by natural disease progression, might help explore the effect of ChEIs on different BPSD. Novel or repositioned drugs such as mibampator, dextromethorphan/quinidine, and citalopram are being tested for the management of agitation in ongoing Phase II and III trials [36,37,38].

There are several limitations in this review. The internal validity may be limited as the methodologies adopted by the included studies differed. Only one of the included studies was a double-blinded randomized controlled trial. Studies with open-label or single blinded designs are influenced by the placebo effect and confirmation bias. Also, only a few clinical trials that studied the effect of ChEIs in the Chinese population are available for analysis. Population size in each study was relatively small, undermining the power of this analysis. Marked heterogeneity, which could be attributed to different disease entity, baseline cognition, medication, and length of follow-up, rendered meta-analysis impossible. There is a potential of publication bias, but the number of studies is too small for testing funnel plot asymmetry [21]. Finally, newer studies that are published after 2015 have not been included in this analysis.

## 5. Conclusions

ChEIs may be effective in improving cognition in Chinese patients with dementia, but their effect on BPSD remains unclear. Studies of ChEIs in Chinese patients are scarce and their quality is unsatisfactory. Further clinical investigation of the interaction between ChEIs and specific neuropsychiatric symptoms, together with basic science research on the mechanisms of subsets of BPSD are warranted.

## Figures and Tables

**Table 1:** Characteristics of studies included in systematic review.

First Author (Year)	Country	Study Design	Intervention	Treatment Duration, Length of Follow-up	Study Population		Mean Age		Baseline Cognition (MMSE)		Baseline BPSD (NPI)	
					Treatment	Control	Treatment	Control	Treatment	Control	Treatment	Control
Chu (2007) [14]	Hong Kong	Cohort study	Galantamine Week 1–4: 4 mg twice daily Week 5–8: 8 mg twice daily Week 5–24 month: 12 mg twice daily or 8 mg twice daily depending on tolerability	2 years, 2 years	42	19	78.5	78.9	14.9 ^a^	16.1 ^a^	11.0 ^a^	7.4 ^a^
Mok (2007) [13]	Hong Kong	RCT	Rivastigmine Week 1–4: 1.5 mg twice daily Week 5–26: 3.0 mg twice daily	26 weeks, 26 weeks	20	20	75.7	74.1	13.0 ^a^	13.4 ^a^	15.0 ^a^	10.6 ^a^
Peng (2005) [23]	China	RCT	Donepezil 5 mg once daily	12 weeks, 12 weeks	46	43	72.6	71.8	17.8 ^b^	18.2 ^b^	Not provided	
Fuh (2004) [22]	Taiwan	Longitudinal observational study	Any ChEIs between follow-up No exposure to ChEIs	Mean = 1.6 years (range: 0.1–3.1 years), mean = 29 months (range: 3–109 months)	194	171	73.1	73.6	16.9	12.0	Not provided	

^a^ *p* > 0.05, ^b^ *p*-value not provided, RCT, randomized controlled trial; ChEIs, cholinesterase inhibitors; MMSE, Mini-Mental State Examination; BPSD, Behavioral and Psychological Symptoms of Dementia; NPI, Neuropsychiatric Inventory.
